# Supplementary figures and images for: High-Resolution Transcriptome Atlas and Improved Genome Assembly of Common Buckwheat, Fagopyrum esculentum
Source: Front Plant Sci. 2021 Mar 16;12:612382. doi: 10.3389/fpls.2021.612382 (PMC8010679; doi:10.3389/fpls.2021.612382)

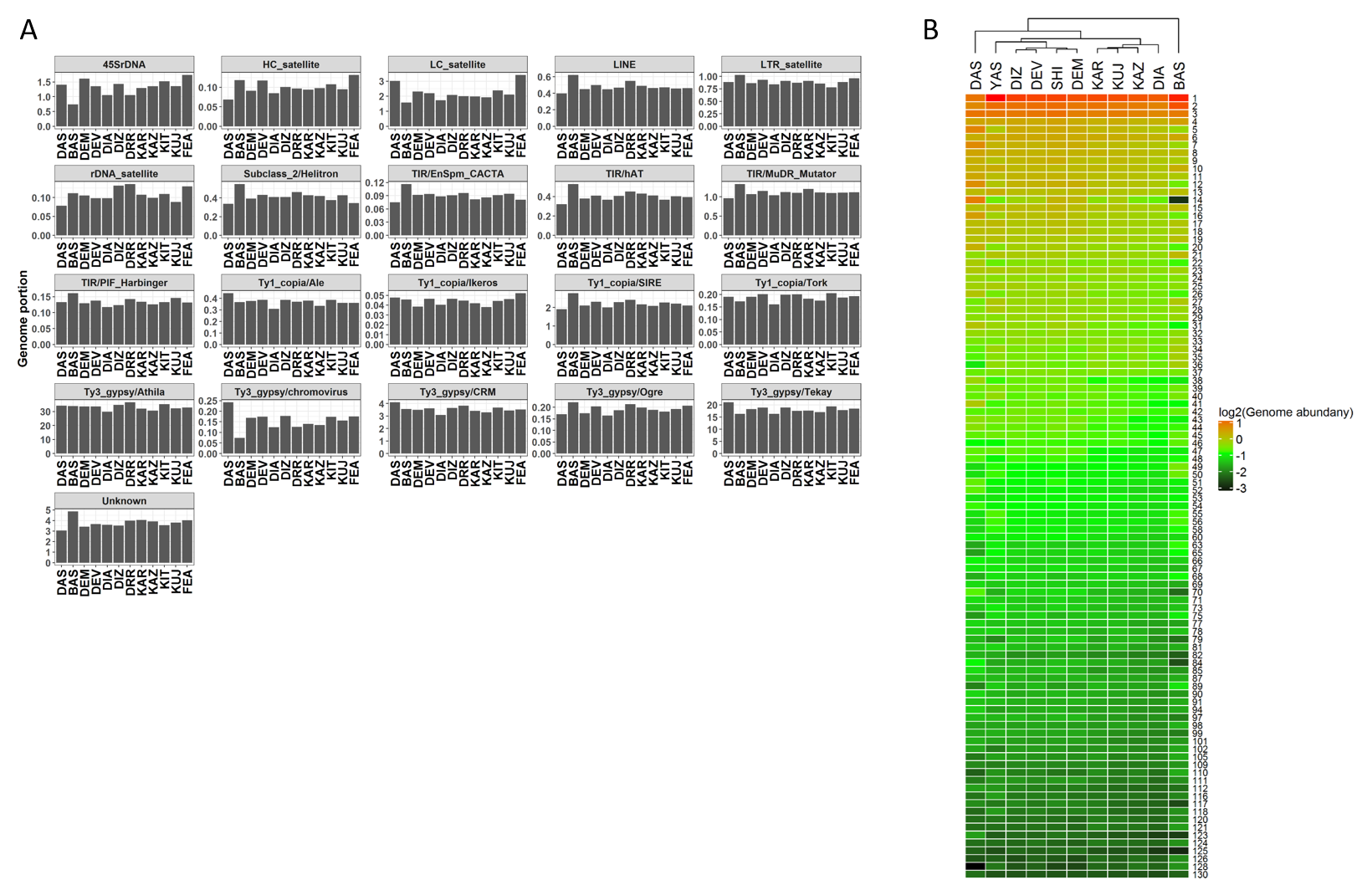

Supplement: Supplementary Figure 1 — Repeat content in F. esculentum spp. ancestrale and different buckwheat cultivars. DAS, Dasha; BAS, Bashkirskya krasnostebelnaya; DEM, Demetra; DEV, Devyatka; DIA, Dialog; DIZ, Dizajn; KAR, Karadag; KAZ, Kazanka; KUJ, Kujbyshevskaya; DRR/YAS, the sample from the study by Yasui et al. (2016), cultivar unknown, presumably of Japanese origin, KIT/SHI, Shinanonatsusoba, FEA – F. esculentum spp. ancestrale. [file Image_1.png]

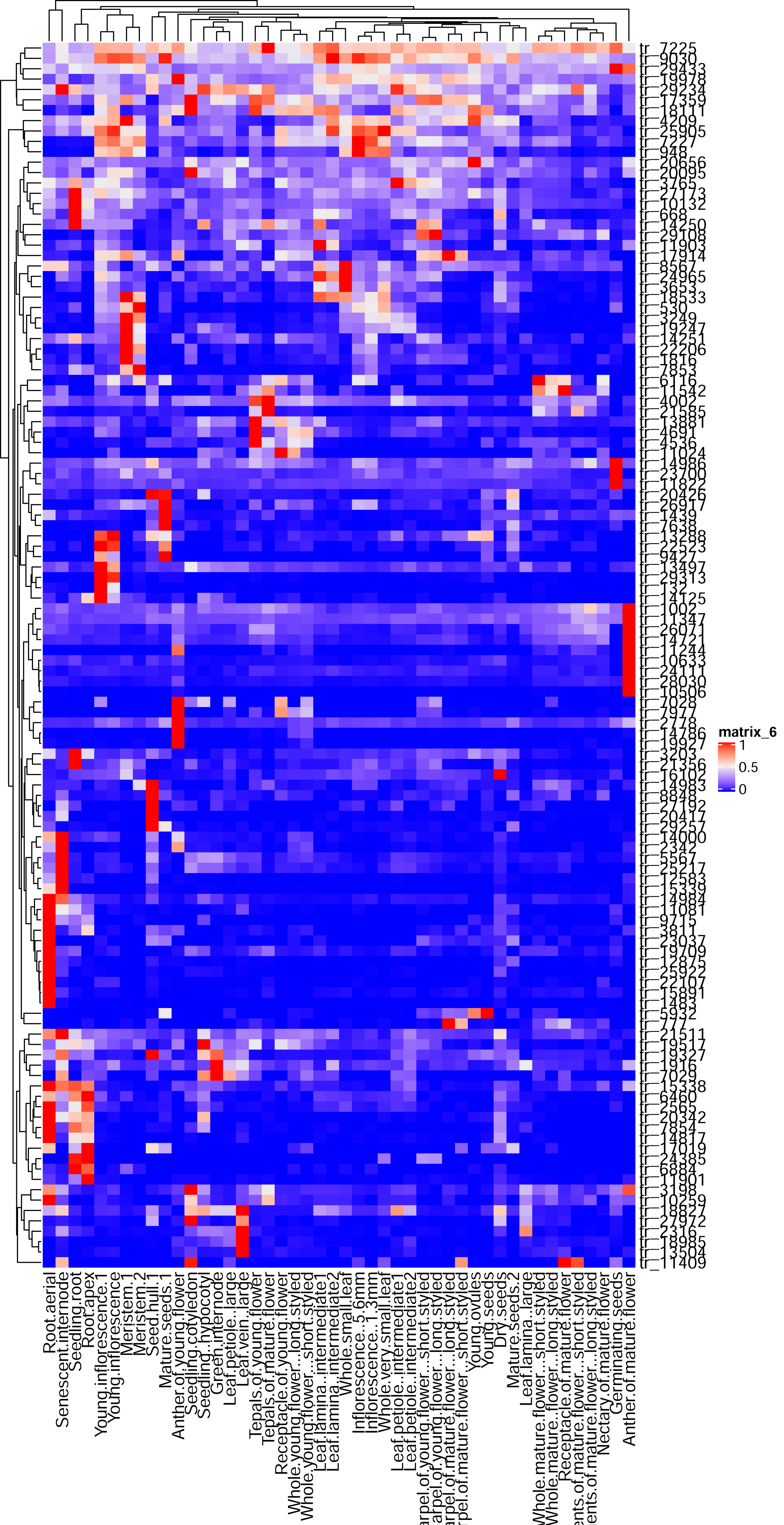

Supplement: Supplementary Figure 2 — The distribution of Shannon entropy across expressed genes of F. esculentum. [file Image_5.png]

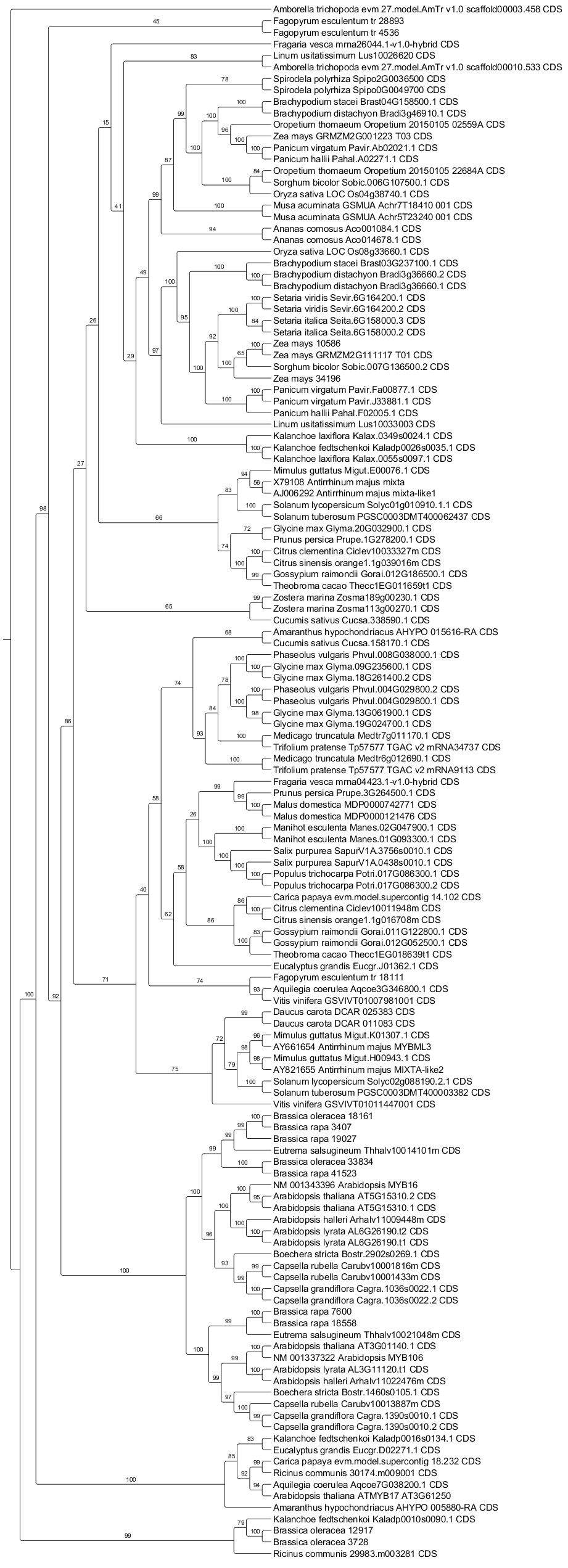

Supplement: Supplementary Figure 3 — Heatmap showing tissue-specific (Shannon enthropy cut-off 0.25) buckwheat genes. For each gene expression values were normalized by maximal value. [file Image_6.jpeg]

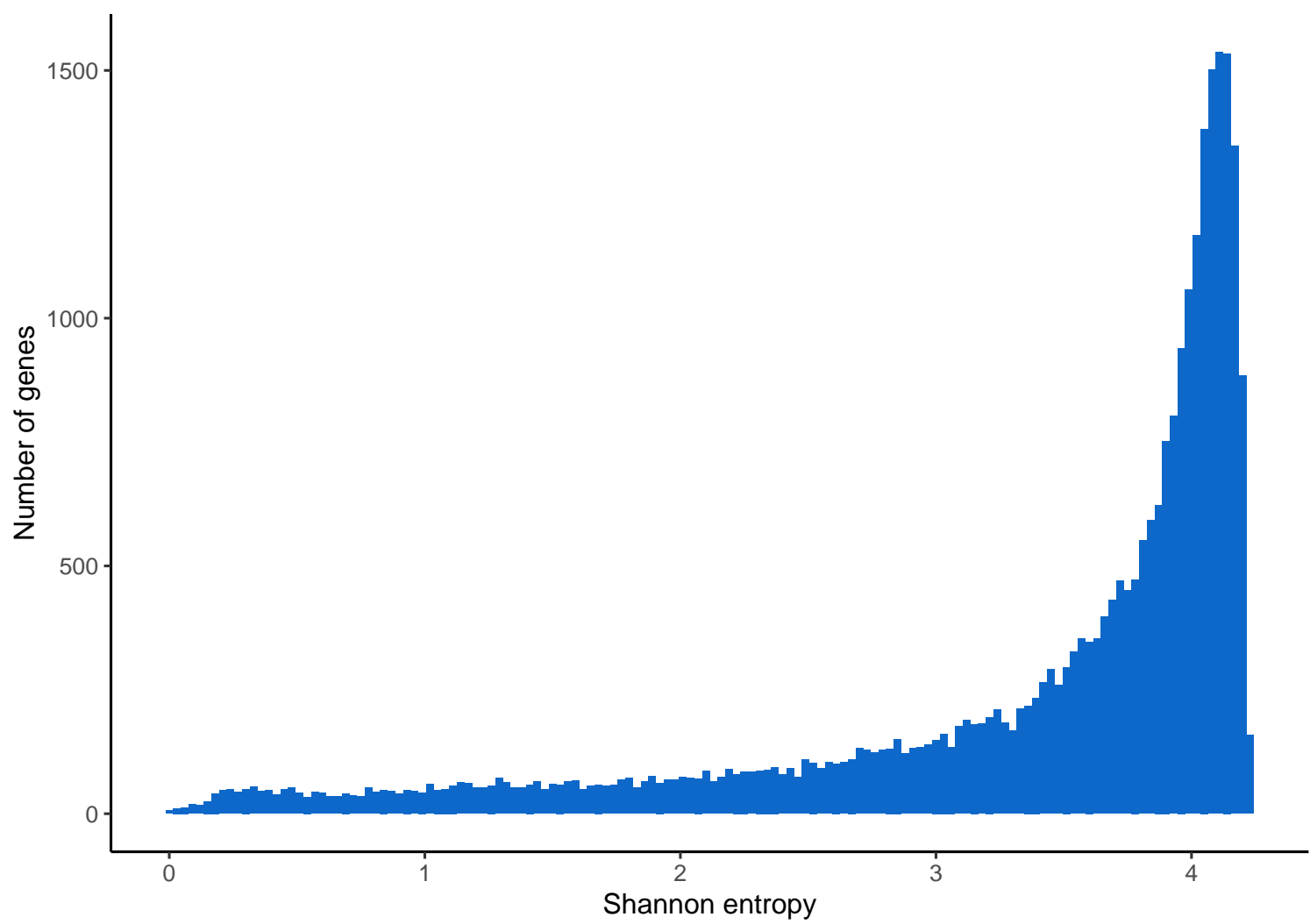

Supplement: Supplementary Figure 4 — Heatmap showing the expression profiles of the genes – orthologs of the stably expressed genes in Arabidopsis thaliana. [file Image_2.pdf]

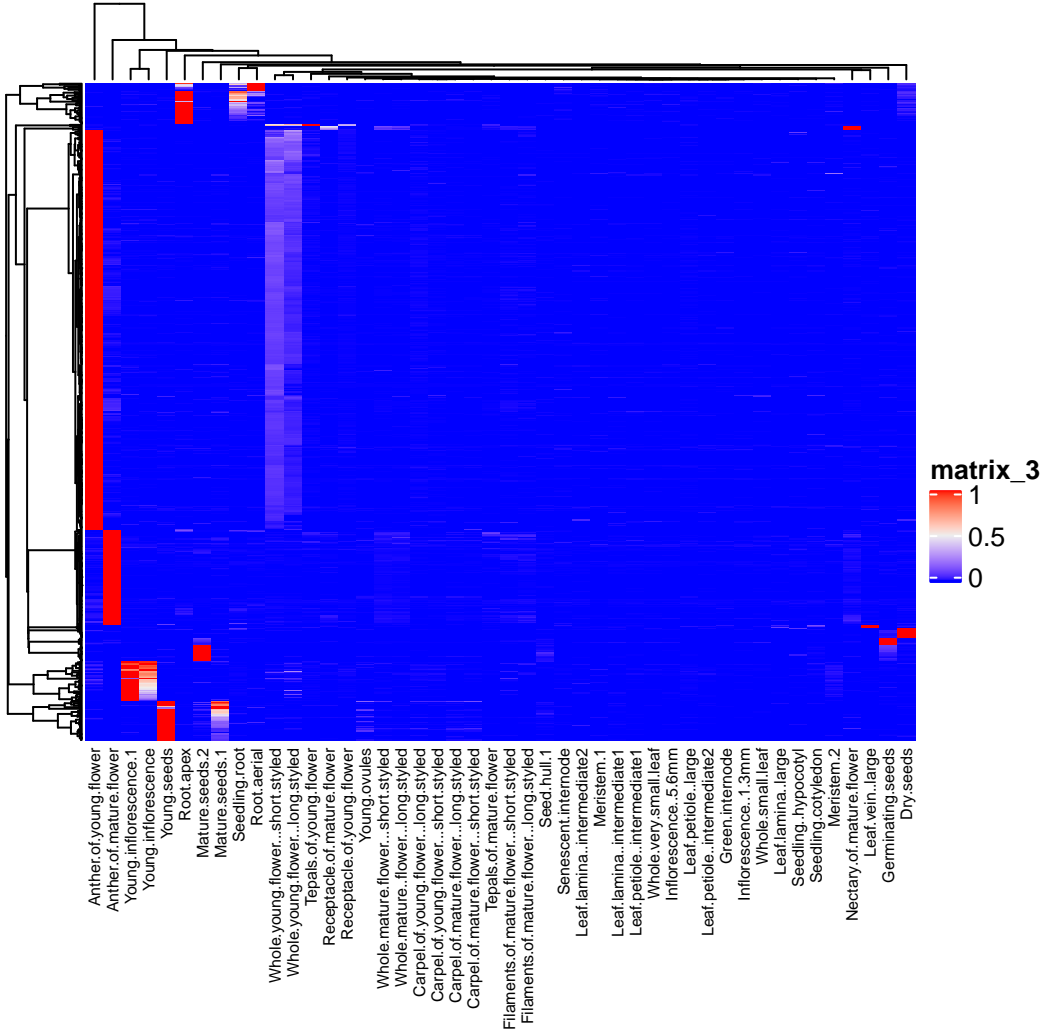

Supplement: Supplementary Figure 5 — Heatmap showing the expression of buckwheat MYB genes sharing significant similarity with MIXTA/MYB16/MYB106. [file Image_3.pdf]

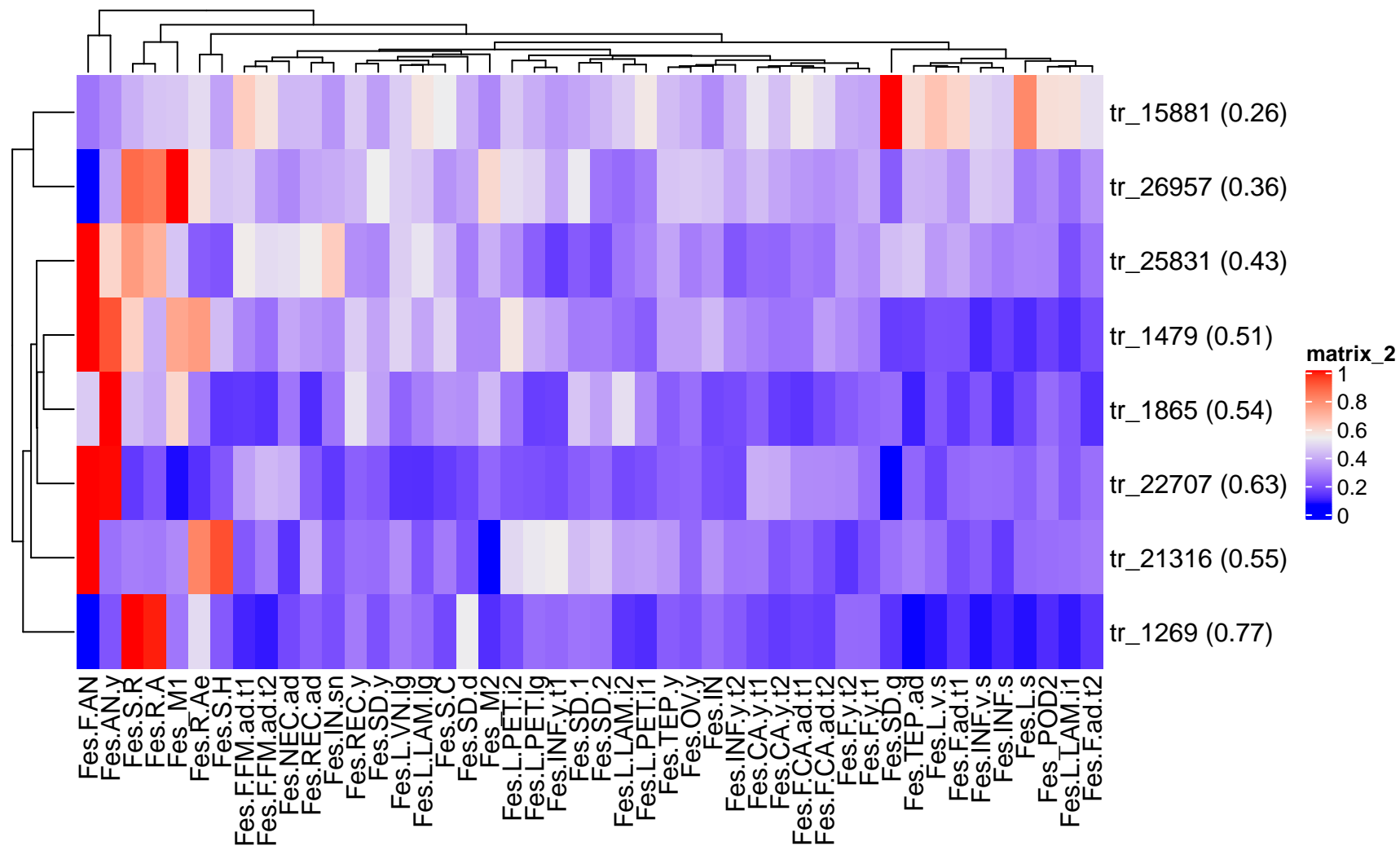

Supplement: Supplementary Figure 6 — Phylogenetic tree of angiosperm MIXTA/MIXTA-like genes. [file Image_4.pdf]
